# Primary prevention of gestational diabetes for women who are overweight and obese: a randomised controlled trial

**DOI:** 10.1186/1471-2393-13-65

**Published:** 2013-03-13

**Authors:** Cate Nagle, Helen Skouteris, Heather Morris, Alison Nankervis, Bodil Rasmussen, Peter Mayall, Richard L Kennedy

**Affiliations:** 1 School of Nursing and Midwifery, Deakin University, Geelong Waterfront campus, Locked bag 20000, Geelong, Victoria 3220, Australia; 2 School of Psychology, Burwood campus, 221 Burwood Hwy, Burwood, Victoria 3125, Australia; 3 Royal Melbourne Hospital, Melbourne, Australia; 4 School of Nursing and Midwifery, Deakin University, 221 Burwood Highway, Burwood 3125, Victoria, Australia; 5 Department of Obstetrics and Gynaecology, Barwon Health, Ryrie St, Geelong 3215 Victoria, Australia; 6 School of Medicine, Deakin University, Geelong Waurn Ponds campus, 75 Pidgons Road Waurn Ponds, Geelong, Victoria 3216, Australia

**Keywords:** Gestational diabetes, Obesity, Prevention, Gestational weight gain, Primary care, RCT

## Abstract

**Background:**

Gestational Diabetes Mellitus (GDM) has well recognised adverse health implications for the mother and her newborn that are both short and long term. Obesity is a significant risk factor for developing GDM and the prevalence of obesity is increasing globally. It is a matter of public health importance that clinicians have evidence based strategies to inform practice and currently there is insufficient evidence regarding the impact of dietary and lifestyle interventions on improving maternal and newborn outcomes. The primary aim of this study is to measure the impact of a telephone based intervention that promotes positive lifestyle modifications on the incidence of GDM. Secondary aims include: the impact on gestational weight gain; large for gestational age babies; differences in blood glucose levels taken at the Oral Glucose Tolerance Test (OGTT) and selected factors relating to self-efficacy and psychological wellbeing.

**Method/design:**

A randomised controlled trial (RCT) will be conducted involving pregnant women who are overweight (BMI >25 to 29.9 k/gm^2^) or obese (BMI >30 kgm/^2^), less than 14 weeks gestation and recruited from the Barwon South West region of Victoria, Australia. From recruitment until birth, women in the intervention group will receive a program informed by the Theory of Self-efficacy and employing Motivational Interviewing. Brief ( less than 5 minute) phone contact will alternate with a text message/email and will involve goal setting, behaviour change reinforcement with weekly weighing and charting, and the provision of health information. Those in the control group will receive usual care. Data for primary and secondary outcomes will be collected from medical record review and a questionnaire at 36 weeks gestation.

**Discussion:**

Evidence based strategies that reduce the incidence of GDM are a priority for contemporary maternity care. Changing health behaviours is a complex undertaking and trialling a composite intervention that can be adopted in various primary health settings is required so women can be accessed as early in pregnancy as possible. Using a sound theoretical base to inform such an intervention will add depth to our understanding of this approach and to the interpretation of results, contributing to the evidence base for practice and policy.

**Trial registration:**

This trial is registered with the Australian New Zealand Clinical Trials Registry (ANZCTR): ACTRN12613000125729

## Background

The prevalence of Gestational Diabetes Mellitus (GDM) in Australia has been reported ranging from 1.7 to 9.6% [1-4] and it has been associated with significant rates of maternal and perinatal complications. Being overweight (a body mass index (BMI) of >25 kg/m^2^) or obese (BMI of >30 kg/m^2^) are recognised risk factors for developing GDM in pregnancy [5-8] and confer a 1.3 to 4.8 increased likelihood of developing this condition [6,8]. Maternal complications of GDM include an increased risk of developing Type 2 diabetes, metabolic syndrome and cardiovascular disease [5,9,10]. Newborns of pregnancies affected by GDM are at risk of macrosomia, shoulder dystocia, birth injuries and hypoglycaemia [11-13]; as young children they are at risk of becoming overweight, and in adolescence of impaired glucose tolerance and obesity [14].

In addition to developing GDM, women who are obese are at increased risk of many pregnancy related conditions including miscarriage, pre-eclampsia, hypertension, infection and thromboembolic disease [11,15-17] and have a greater chance of caesarean section [11,15,16,18-21], induction of labour [15,16,18,21] and death related to childbearing [11,19,21-23].

Compared to babies of women in a normal weight range, babies of overweight and obese women are more likely to be macrosomic [12,15,16,22,24], preterm [11-13,16,25], have a congenital anomaly [11-13,24], be admitted to the neonatal intensive care unit [11-13,25] and need treatment for jaundice or hypoglycaemia [11,16,17]. Thus, GDM particularly for those overweight and obese, increases both short and long term risks to the health of both the woman and her baby.

Over a third of pregnant women in Australia are either overweight or obese [2,6,11] and prevalence is higher in rural and regional areas than in metropolitan areas [6,26]; a prevalence of overweight and obesity of 65.5% has recently been reported in the pregnant population of north-east Victoria [6]. The rates of obesity are also increasing internationally [27] and identification of evidence based strategies to reduce the incidence of GDM and promote more healthy lifestyle choices in women who are overweight and obese needs to be prioritised to inform practice and health policy.

Healthy weight management for overweight and obese women includes avoiding excessive weight gain [28] and ideally aligning gestational weight gain to recommended ranges based on BMI [29-31]. However, intervention studies [32-35] have reported only a third to a half of obese women achieve a gestational weight gain within the recommended range [36,37]. Furthermore, there is limited evidence to support the effectiveness of dietary and physical activity lifestyle interventions in preventing adverse perinatal outcomes for obese women. In a systematic review of nine trials involving 743 women, Dodd and colleagues [38] concluded that the effectiveness of lifestyle interventions in pregnancy remains unclear. This is of significant concern and forms the rationale for trialling a different approach to preventing excessive weight gain in obese women.

There is a paucity of robust evidence to guide practice, with a limited number of published randomised controlled trials that have tested interventions with prevention of GDM as the primary outcome [39-41]. One of these trials [40], tested the effectiveness of a composite intervention versus usual care in preventing GDM. The intervention incorporated elements of continuity of care, measuring weight gain at each clinic visit, a regular five minute visit with a food technologist and a session with a clinical psychologist. The researchers concluded that the repetition of the intervention contributed to the positive outcomes. However, the intensive nature of the intervention, with a reliance on specialised staff, raises concerns regarding accessibility and sustainability in low socioeconomic status (SES) settings particularly rural and regional health care services.

Based on the approach of Social Cognitive Theory [42] and using the principles of Motivational Interviewing [43] we have developed a composite intervention that employs continuity of contact through weekly communication with the participating pregnant woman. The EDGE intervention (Educate, Develop Goals, Engage) modifies elements of Quinlivan et al. [40], specifically continuity of contact and reinforcement of behaviour modifications, and applies these to a format that can be trialled in areas of low SES and more broadly. The EDGE program will commence at recruitment and continue until birth.

We propose to explore the effectiveness of the EDGE intervention compared to usual care, in preventing the incidence of GDM in overweight and obese women. The results of this trial will contribute to our understanding of how to effectively facilitate healthy gestational weight gain in pregnancy, particularly in areas where there are issues of access to health care resources.

## Methods

This study will be conducted in the primary care setting of Barwon South West, Victoria, Australia. A RCT design will be employed where pregnant women will be individually randomised and allocated to either the intervention or a control group. The study will be conducted and reported using the CONSORT recommendations [44] (Additional file 1).

### Aims

The primary aim of this trial is to measure the impact of the EDGE intervention compared to usual care on the incidence of GDM in overweight and obese women. The primary hypothesis is that women randomised to the EDGE intervention will have 10% fewer diagnoses of GDM than women in the control group. There are four secondary aims: to measure the impact of the EDGE intervention compared to usual care on 1) restricting gestational weight gain to the weight ranges recommended by the Institute of Medicine [29]; 2) reducing the incidence of babies born large for gestational age (LGA); 3) differences in blood glucose levels taken at the Oral Glucose Tolerance Test (OGTT) and 4) promoting self-efficacy and selected factors for psychological well-being.

### Participants

Inclusion criteria: women with a singleton pregnancy will be included if their gestation is less than 14 weeks and their BMI ≥25. Exclusion criteria will include women: with pre-existing diabetes (types 1 and 2), a past history of gestational diabetes, unable to give informed consent in English, currently experiencing vaginal bleeding or with severe medical conditions preventing them from being able to undertake regular low impact exercise.

### Recruitment strategies

Women will be recruited from 30 participating general practices within the rural and regional area surrounding Geelong, Victoria. All women who meet the selection criteria will be informed of the study by the general practitioner (GP) or Practice Nurse (PN). Women interested in participating will be provided with a Participant Information and Consent Form and the GP/PN will obtain written informed consent and complete an enrolment form; both documents will be forwarded to the research office. All women enrolled in the trial will be given a study pack by the recruiting GP/PN that contains the first questionnaire with a reply paid envelope and a copy of the consent form.

### Randomisation procedure

A computer generated randomisation sequence will be used to facilitate the block randomisation of participants. Upon receipt of the consent and enrolment form by the research office, random allocation to the intervention or control group will be conducted by an individual who is independent to the study by drawing opaque numbered envelopes consecutively, to reveal the group allocation.

All women will receive written health information: 1) following recruitment and a reminder/thank-you for returning the questionnaire (Additional file 2) and 2) with a reminder/thank-you for returning the second questionnaire (Additional file 3). Both resources are used in clinical practice and are readily available.

Women allocated to the intervention group will receive the EDGE program from recruitment until birth, consisting of weekly contact with a brief (five minute) phone call each fortnight that employs Motivational Interviewing techniques on topics such as: identifying barriers and facilitators to positive behaviour change; encouraging goal setting; reinforcing positive lifestyle change; promoting self-monitoring and charting of weight gain and reflecting on behaviour change. The phone will alternate each week with text message/email contact to engage, support and reinforce behaviour change.

Women in the control group will receive usual care. Usual care in this setting involves initial weighing to calculate BMI [30]. There will be no change to clinical care beyond the ascertainment of weight from 36 weeks.

A reminder regarding weighing will be sent to all women with the second questionnaire.

### Blinding

All participating women will provide written consent prior to randomisation however women will not be blinded to allocation. Due to the type of intervention, blinding of the researchers to allocation is also not realistic. Analysis will be performed by a person independent to the research team to avoid assessment bias.

### Primary outcome and measures

#### Gestational diabetes

The primary outcome is the proportion of women diagnosed with GDM between 24 – 28 weeks and will be defined using both the Australasian Diabetes in Pregnancy Society (ADIPS) management guidelines and the International Association of Diabetes and Pregnancy Study Groups (IADPSG) criteria. The use of different measures reflects the current controversy that exists in practice [3] and both measures will be reported.

Both ADIPS and IADPSG use a 75 gram glucose load. Using ADIPS criteria, GDM is diagnosed with a fasting plasma glucose level ≥5.5 mmol/L or a 2 hour level ≥8.0 mmol/L [45]. The IADPSG criteria diagnose GDM if any of the following levels are reported: a fasting plasma glucose level ≥5.1 mmol/L; a 1 hour level ≥10 mmol/L or a 2 hour level ≥8.5 mmol/L [46].

### Secondary outcomes and measures

#### Gestational weight gain

Differences in the proportions of women with gestational weight gain within the IOM (Institute of Medicine) guidelines [29] will be compared. Weight gain will be calculated as the difference between the booking-in weight and the last recorded weight from 36 weeks gestation to birth, as recorded in the pregnancy care records. IOM guidelines recommend women with BMI ≥25 to 29.9 kg/m^2^(overweight) gain 7–11.5 kilograms and women with a BMI ≥30 kg/m^2^ (obese) gain 5–9 kilograms [29].

### Large for gestational age (LGA) infants

The differences in the proportions of babies that are LGA will be compared. LGA infants will be defined in keeping with other trials [38] as a birth weight ≥90^th^ centile for gender and gestation, as well as a birth weight > 4000 grams. These data will be obtained from a review of medical records and will be reported using both criteria.

### Psychological health

Validated scales, where available, will be used to measure self- reported psychological wellbeing and engagement in behaviour change: anxiety; depression; self- efficacy and readiness to change behaviour will be measured at enrolment and at 36 weeks gestation using questionnaires in hard copy.

### Anxiety

Anxiety will be measured using the short version of the Speilberger State-Trait anxiety inventory (STAI-State) [47]. Respondents use a four point scale for six items to indicate how they feel now (1 = Not at all to 4 = Very much). With reversing of specific responses, mean scores are reported and higher scores indicate greater anxiety.

### Depression

Depression will be measured using the Beck Depression Inventory II [48] a 21 item scale with respondents selecting from one of four statements. Each response is assigned a score of 0 to 3 and the total score is the sum of all responses. This scale has been validated to use in pregnancy; a cut off >16 affords a sensitivity of 0.83, a specificity of 0.89, positive predicted value of 0.50 and negative predictive value of 0.98 PPV [49]. The mean differences between groups will be compared.

### Self-efficacy

Self-efficacy related to healthy lifestyle changes in diet and exercise will be measured and the mean differences between groups will be compared. Self-efficacy related to eating will be measured using a 20 item, 10 point scale ranging from 0 (not confident) to 9 (very confident) [50]. Sub-scales of the Weight Efficacy Life-Style Questionnaire include: negative emotions, availability, social pressure physical discomfort and positive activities; the total score is the sum of all items. The Cronbach alpha coefficients of internal consistency ranged from 0.70 for positive activities to 0.90 for social pressure [50].

The Self-Efficacy for Exercise Scale will also be used and consists of nine items that respondents score from 0 (not confident) to 10 (very confident) [51]. The Cronbach alpha coefficient of internal consistency is 0.92 and reliability using the squared multiple correlation coefficient ranged from 0.38 to 0.76 [51].

### Readiness to change

Readiness to change, the importance of change and confidence in changing will be measured using the Readiness to Change Questionnaire [52] which researchers have modified for the weight loss context. This scale measures stages of change (pre-contemplation, contemplation and active) using 12 items each with a five point rating scale from 1 (strongly agree) to 5 (strongly disagree). Information from this scale will be used descriptively.

### Differences in plasma glucose levels

Differences in mean values of fasting, 1 hour and 2 hour plasma glucose levels from the OGTT will be compared using both ADIPS and IADPSG criteria in both groups.

### Economic evaluation

Resource use data will be collected from medical records and projections of health cost savings will be informed by pregnancy and birth outcome data.

### Power, sample size and retention

The primary outcome is the proportion of overweight and obese women diagnosed with GDM. The proportion of obese women diagnosed with GDM is 17% in a Victorian context [40]; a difference 10% effect size is considered clinically significant. It is estimated that 370 women are required (185 per study arm) in order to detect a difference of 10% between groups with 80% power at a 0.05 level of significance. With an approximate expected participation of 77% [53] and attrition rate of 15% [53] the required sample of 370 women will be recruited.

### Data collection

A record review from the local hospitals will be conducted after birth to provide data for GDM and gestational weight gain outcomes. Socio-demographic data, pregnancy and birth data will be collected from the medical records and used descriptively to interpret findings. Data will also be obtained from medical records of General Practices if required.

Self-reported baseline and outcome data will be collected using a paper based questionnaire. The initial questionnaire will be provided at recruitment and the second questionnaire will be posted at 36 weeks; reply-paid envelopes will be provided for both questionnaires.

### Ethics

Ethics approval to conduct this study has been provided by the Human Research Ethics Committee (HREC) of Barwon Health 12/108 and the project has been endorsed by Deakin University HREC.

### Analyses

Initial analysis will examine baseline characteristics of all women, as an indication that the treatment groups were comparable for selected variables including, age, parity and BMI category. Primary and secondary outcomes will be analysed on an "intention to treat" basis. Differences between trial arms will use the Chi-squared statistic for categorical outcomes and Student’s t-test for continuous outcomes and 95% confidence intervals will be reported. Appropriate regression models will be employed controlling for co-variants. The fidelity of the intervention will be assessed and reported and the feasibility of the intervention will be considered in the process evaluation that will include the experience of participants and GP/PNs.

## Discussion

GDM is a condition that can impact on the short-term and long term health of both the mother and her baby. The risk factor of obesity is a significant contributor for this condition and the prevalence of obesity, particularly in low SES environments, is increasing. To date there is a lack of evidence to inform practice and policy to reduce the incidence of GDM and gestational weight gain so that birth outcomes are optimised. The findings of this study will inform an area of practice that is of international significance; trialling an intervention that has the potential to be sustainable in the primary care setting and may have broad application.

## Competing interests

The authors declare they have no competing interests.

## Authors’ contributions

CN, HS & HM designed the study, with expert input from AN, PM and RLK. CN developed the first draft of this manuscript and all authors contributed to subsequent drafts. All authors read and approved the final manuscript.

## Pre-publication history

The pre-publication history for this paper can be accessed here:

http://www.biomedcentral.com/1471-2393/13/65/prepub

## Supplementary Material

Additional file 1 **CONSORT Flow Diagram.** Word document presenting the data collection points and reporting study’s flow from recruitment to the reporting of outcomes. Click here for file

Additional file 2 **Nutritional Fitness in Pregnancy pamphlet.** Health information pamphlet (also accessible online) given to women in both arm of the trial following recruitment. Click here for file

Additional file 3 **Pregnancy Weight Matters pamphlet.** Health information pamphlet (provided as usual care to all women booked at Barwon Health) given to women in both trial arms with a reminder/thank-you for returning the questionnaire at 36 weeks. Click here for file
