# Supplementary material for: Primary prevention of gestational diabetes for women who are overweight and obese: a randomised controlled trial
Source: BMC Pregnancy Childbirth. 2013 Mar 13;13:65. doi: 10.1186/1471-2393-13-65 (PMC3669050; doi:10.1186/1471-2393-13-65)
Supplement: Additional file 1 — CONSORT Flow Diagram. Word document presenting the data collection points and reporting study’s flow from recruitment to the reporting of outcomes. [file 1471-2393-13-65-S1.doc]

**Additional file 1: CONSORT Flow Diagram**

**Allocation**

**Q1**

Booking visit <14 weeks

**Enrollment**

Assessed for eligibility (n= )

Excluded (n= )

♦  Not meeting inclusion criteria (n= )

♦  Declined to participate (n= )

♦  Other reasons (n= )

Allocated to intervention (n= )

♦ Received allocated intervention (n= )

♦ Did not receive allocated intervention (give reasons) (n= )

Allocated to control (n= )

♦ Received allocated control(n= )

♦ Did not receive allocated intervention (give reasons) (n= )

Randomized (n= )

**Analysis**

**Q2**

36 weeks

**Record review**

Analysed (n= )
♦ Excluded from analysis (give reasons)

(n= )

Lost to follow-up (give reasons) (n= )

Discontinued intervention (give reasons) (n= )

Lost to follow-up (give reasons) (n= )

Discontinued intervention (give reasons) (n= )

Analysed (n= )
♦ Excluded from analysis (give reasons)

(n= )
