# Supplementary material for: Primary prevention of gestational diabetes for women who are overweight and obese: a randomised controlled trial
Source: BMC Pregnancy Childbirth. 2013 Mar 13;13:65. doi: 10.1186/1471-2393-13-65 (PMC3669050; doi:10.1186/1471-2393-13-65)
Supplement: Additional file 2 — Nutritional Fitness in Pregnancy pamphlet. Health information pamphlet (also accessible online) given to women in both arm of the trial following recruitment. [file 1471-2393-13-65-S2.pdf]

WOMEN AND NEWBORN HEALTH SERVICE

King Edward Memorial Hospital

Nutritional Fitness in Pregnancy

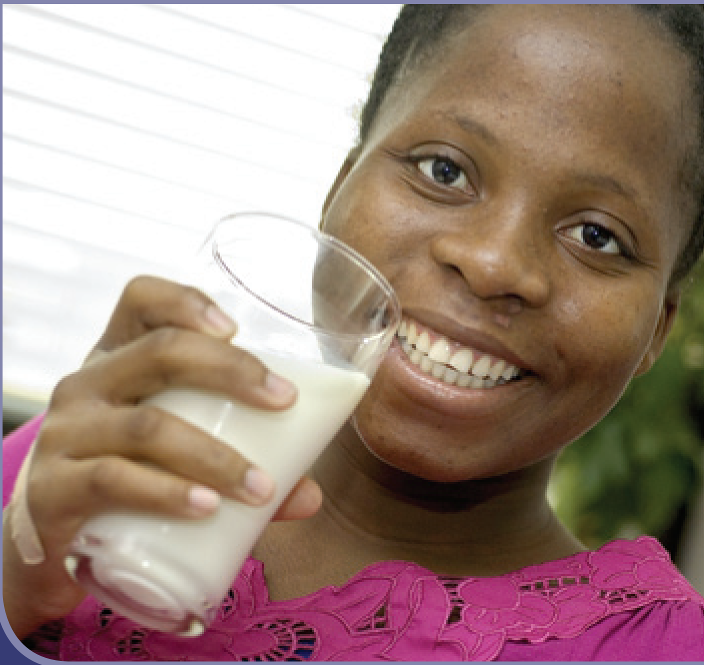

Delivering a **Healthy WA**

Why Fitness?

Because of the demand placed on your body to provide nourishment for a healthy baby, while increasing just about all of your own normal vital functions, including cardiac output and respiration increase.

The ability to respond to these demands will depend partly upon your nutritional fitness.

Conception begins an amazing array of changes in your body. Looking at these changes as they occur in the mother, placenta and fetus throughout pregnancy will show the special needs for nutrients and how important the choice of foods becomes.

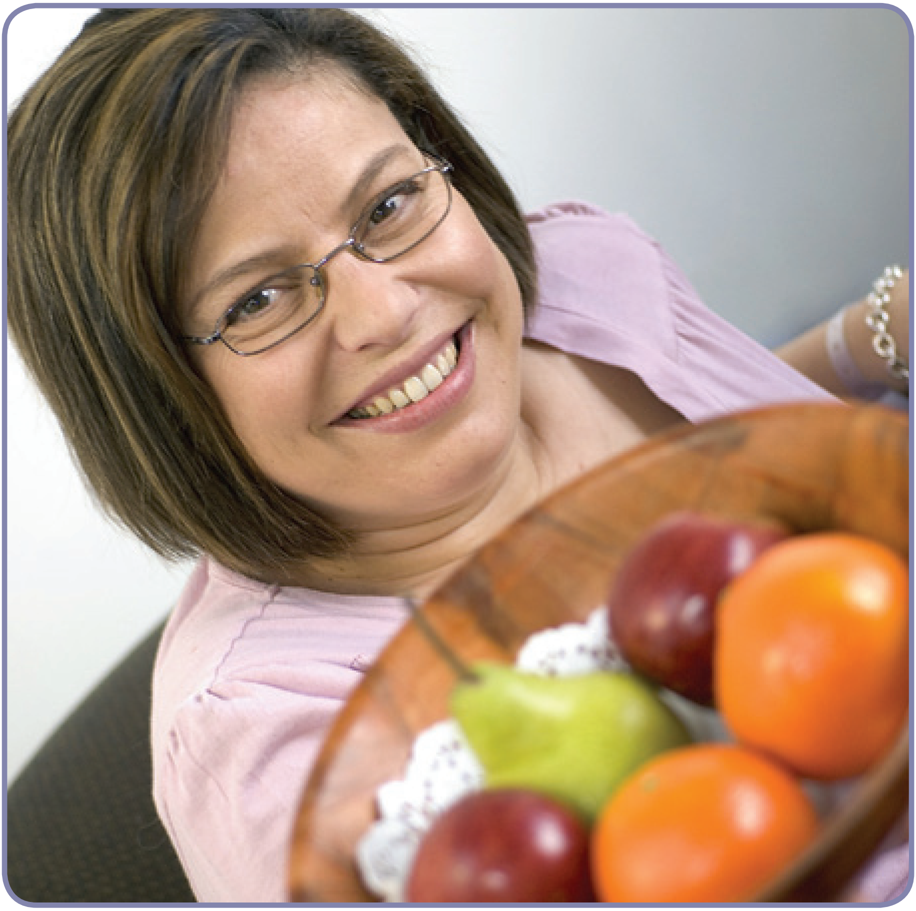

The First Trimester

The changes	Special points about nutrition
• In early pregnancy you may feel tired and experience some nausea and vomiting. ‘Morning sickness’ however may occur at any time of the day.	• Choose smaller, lighter meals more frequently and use plenty of easily digested carbohydrate foods to counteract morning sickness and revitalize.
	**Where do you find carbohydrates?**<br>In breads, breakfast cereals, pasta, rice, crackers, fruits and vegetables.
• Be aware that caffeine can irritate the lining of your stomach.	• Limit caffeine intake to less than 200mg (milligrams) per day.
	**How much caffeine in a cuppa?**<br>Coffee instant 80-100mg/cup<br>percolated 100-150<br>brewed 100-500<br>drip 100-120<br>decaffeinated 2-4<br>Tea - amount depends on strength of the brew 8-90mg/cup<br>Cola drinks 45mg/375ml can<br>Hot chocolate or Cocoa 50-70mg/cup<br>Chocolate bars 30mg/20gm bar
• Your body now uses slightly more energy to maintain its normal functions.	• You do not need to eat more food, but try not to skip meals. Use foods rich in B vitamins so that release of energy in the cells is more efficient.
	B vitamins abound in wholegrain cereals, legumes, lean pork, lean beef and lamb, chicken and fish, leafy green vegetables milk and cheese.

The First Trimester (continued)

<table>
<tr><th>The changes</th><th>Special points about nutrition</th></tr>
<tr><td rowspan="5">• Your blood volume begins to increase in readiness for carrying more nutrients and energy to your growing baby.

• In the first trimester the placenta grows, in size and weight, more than your baby.

• The fertilised egg undergoes rapid cell division and the organs are forming in the first two to eight weeks.</td><td>• A woman's need for folate (folic acid) has doubled. This vitamin is needed for blood formation and building of body cells. You will benefit from a high intake of folate especially if you have used the oral contraceptive pill for a long time.</td></tr>
<tr><td>How can you get more folate?
Highest levels are in fresh green leafy vegetables (raw or lightly steamed) and liver.
Moderate levels in vegemite, marmite, promite, nuts, oranges, avocados and wholegrain bread.</td></tr>
<tr><td>• Protein is the building material for all cell growth. Are you eating two serves of high protein foods each day?</td></tr>
<tr><td>Protein foods are: lean meat, fish, chicken, milk, egg, cheese, also dried beans (like kidney beans), lentils and nuts.
(If you are a vegetarian, refer to 'Vegetarian Pregnancy' pamphlet, available KEMH).</td></tr>
<tr><td>• Alcohol is best avoided. It provides many calories and few nutrients. The placenta allows alcohol through to the baby who cannot break it down properly.</td></tr>
</table>

The First Trimester (continued)

The changes	Special points about nutrition
• The baby's brain, spinal cord and nerves are forming.	• The development of the nervous system requires both polyunsaturated and saturated fats. So while a high fat diet is not recommended, do choose a variety of fats in moderation.
	**Eat less fat!**<br>Use butter or margarine sparingly.<br>Choose fat reduced milk.<br>Cut out fried foods: grill, steam, bake or boil.<br>Trim fat from meat and remove chicken skin before cooking.<br>Cut down your intake of cakes, biscuits, pastries, crisps, greasy takeaways, battered/crumbed food and rich desserts.
	• Eat one to two fish meals per week. Avoid eating swordfish, shark/flake, orange roughy and catfish as these types may have too much mercury. The fetus is vulnerable to the harmful effects of mercury.<br>• Iodine is an essential nutrient for the developing brain. Fish and shellfish are good sources. Use iodized salts.
• Increased progesterone levels cause the smooth muscle in you uterus and gastrointestinal tract to relax: the movement of food through your gut becomes slower allowing more time for nutrient absorption.	• Increasing your fibre intake now will help prevent constipation.

The First Trimester (continued)

The changes	Special points about nutrition
	Eat more fresh fruit and vegetables. Wholemeal and wholegrain breads, cereals, wholemeal pasta and brown rice.
	• Check that your fluid intake is adequate as fibre needs water to be effective. Six to eight glasses of any fluids each day will be enough.
• Your saliva becomes more acidic and greater care should be taken to avoid tooth decay.	• Sugary drinks and sweet foods provide little nutrient value and greatly increase your risk of tooth decay.
	**Eat less sugar!**<br>Cut down on the amount of sugar you add to drinks and food. Avoid sweets, soft drinks, cordial and flavoured milk, along with sweet cakes and biscuits. Cut out chocolates, lollies, honey and jam.<br>Low joule soft drinks and cordials sweetened with nutra sweet and Equal sweetener are all safe to use in pregnancy.
• You can expect a weight gain of one to two kilograms in this trimester. An early more rapid weight gain is often characteristic of multiple pregnancy or catch-up growth if you are underweight.	• There is no increase in energy (calories/kilojoules) required in the first trimester, but food choices should be changing in preparation for the high nutrient demands of later pregnancy

The Second Trimester

This stage of the pregnancy, from 13 to 26 weeks, is a time of great change. The mother, placenta and fetus are three parts of a unique relationship, working together with each part strengthening and making possible the actions of the others.

The changes	Special points about nutrition
• You can expect a weight gain of a little less than half a kilogram each week to allow for the growth of your baby and the storage of a fat reserve for later in pregnancy and breastfeeding.	• The amount of energy needed from food now increases by 10-15%. This represents approximately 100-150 calories.<br>• Nutrient needs in pregnancy are greater than energy needs, so improve the quality and variety of your food choices while eating only a little more. For example, you need 200% more iron and 50% more calcium. (For more detailed information see the 'High Iron Foods' and 'High Calcium Foods' pamphlets available at KEMH). Continue to avoid high fat and high sugar foods so you will be able to increase nutrients without adding lots of calories.
• Your taste may change and you may experience an increase in appetite.	• A pregnant women should 'eat to appetite' but not take this as an opportunity to overeat or to indulge in junk food.
• Your blood volume increases in order to supply your baby with oxygen and nutrients as well as give you a healthy glow.	• It is not necessary to include vitamin and mineral supplements if your food choices mirror those suggested in the 'Food Wheel' on page 10.

The Second Trimester (continued)

The changes	Special points about nutrition
• The placenta is now an active organ, able to concentrate some nutrients from your blood to ensure that your baby is well supplied.	• A high calcium diet will ensure that the calcium stores in your bones are not depleted.<br><br>**How much calcium is enough?**<br>A pregnant women needs 1100mg/day.<br>For example<br>2 cups HiLo milk 700mg<br>1 cube cheese 260mg<br>1 orange 50mg<br>1 large cup broccoli 160mg<br>TOTAL 1170mg<br>(For more information see: 'High Calcium Foods': Available from KEMH)
• At 20 weeks gestation, your baby is growing in length, at a maximum rate.	• Your skin needs ten minutes in the sun a day (avoid 10.00am - 2.00pm or 11.00am - 3.00pm during daylight saving hours). This helps you make vitamin D which is needed for bone development.
• Your body conserves the simple sugar glucose for your baby as its growth and movement increases. Your baby will often move noticeably more after you have eaten carbohydrates.	• Glucose is best supplied by complex carbohydrate foods (breads, breakfast cereals, pasta, rice, vegetables) and by the natural sugars in fruit and milk. Remember, you should not skip meals.

The Third Trimester

The developing fetus enters this trimester 35cms long, weighing 0.9kg on average, but will grow into a 51cm long, 3.4kg baby. The third trimester is characterised by growth: muscles gain in bulk and strength; nerve fibres function more efficiently and bones develop and add strength. Optimal nutrition will be rewarded with a healthier less stressful final three months for mother and baby.

The changes	Special points about nutrition
• Around the 30th week of pregnancy, you reach maximum blood volume. On average you have 50% more blood.	• Increasing both the content and your body's absorption of iron from food will prevent anaemia.
• The baby accumulates most of its iron stores in the last trimester, and can do so at the mother's expense.	**Where can you get iron?**<br>Highest levels are in red meats, chicken and fish. Other foods like wholegrain cereals and breads, dried fruits, broccoli and spinach, will provide more iron if eaten at the same time as either meat or foods high in vitamin C (eg orange, capsicum, tomato or fresh juices).<br><br>(For more information, see 'High Iron Foods' available from KEMH).
• Most of the minerals must be supplied to the baby now to strengthen bones and form teeth.	• Your baby will draw the calcium it needs from the 'calcium pool' in your bones. It is important that you have a high intake of calcium at this time.

The Third Trimester (continued)

The changes	Special points about nutrition
• Your weight gain will continue to average half a kilogram each week. A total weight gain between 9 and 13 kilograms is considered optimal. There will be quite healthy variations around this average: a women commencing pregnancy underweight, should gain the upper value, perhaps more; whereas an overweight women would do better to gain only six kgs.	• Unsupervised dieting, fasting and restriction of food intake is **not** recommended. There is some evidence that the mother can protect her own fat stores at the expense of the baby's weight.
• Excessive weight gained in pregnancy may be difficult to lose after delivery and does carry some risks.	• The time to diet is either before or after pregnancy.
• Heartburn may occur more often as your baby grows because the pregnancy hormones cause relaxation of the muscle at the top of your stomach.	• Small meals taken more frequently will help. Avoid caffeine containing drinks as they irritate your stomach lining. Cut out fried and fatty foods, as well as strongly flavoured or spiced foods. Taking iron tablets can make heartburn worse. Check with your doctor.
• Your baby accumulates a remarkable amount of fat in the last weeks of your pregnancy. At 34 weeks gestation, your baby's weight increases at its greatest rate.	• Eat regular balanced meals so that all the nutrients are at optimum levels in your blood and glucose is in constant supply for baby's energy needs.

The Third Trimester (continued)

The changes	Special points about nutrition
• Constipation occurs more frequently.	• Fefol and other iron supplements can cause heartburn, constipation and/or diarrhoea. Dietary changes can provide both relief of these symptoms and a reduced need for oral iron supplements.
• Near delivery higher levels of the maternal hormone estrogen results in a higher water content of your body tissues. This is noticeable as fluid retention ('puffiness') in your face, fingers and feet.	• Women should not severely restrict water or salt. There is enough sodium and other mineral salts present in the large variety of foods recommended for pregnancy without the addition of cooking or table salt. Moderate use of added salt is acceptable.

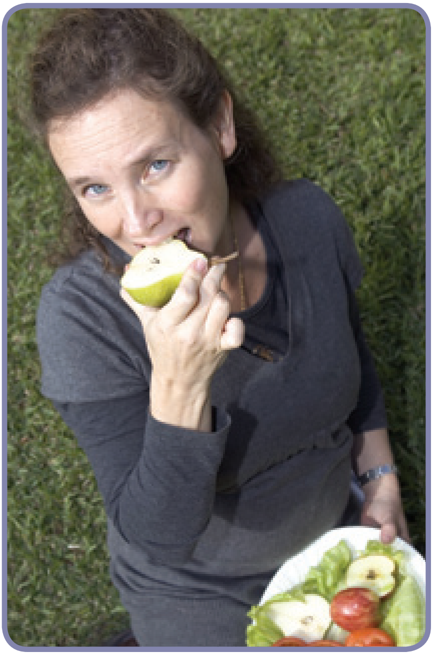

Food wheel

A pattern for daily food choices in pregnancy

BREADS AND CEREALS
1 serve is:
- 1 slice bread
- 2 large/4 small crackers
- 1/2 cup cooked rice
- 1/2 cup cooked pasta
- 2/3 cup breakfast cereal
- 1/2 muffin
- 1/2 bagel

FRUITS
1 serve is an average piece of whole fruit
- a melon wedge
- 180mls fruit juice
- 1/2 cup berries, sliced or cooked fruit
- 1/4 cup dried fruit

VEGETABLES
1 serve is 1/2 cup cooked or raw, or 1 cup of raw leafy vegetables

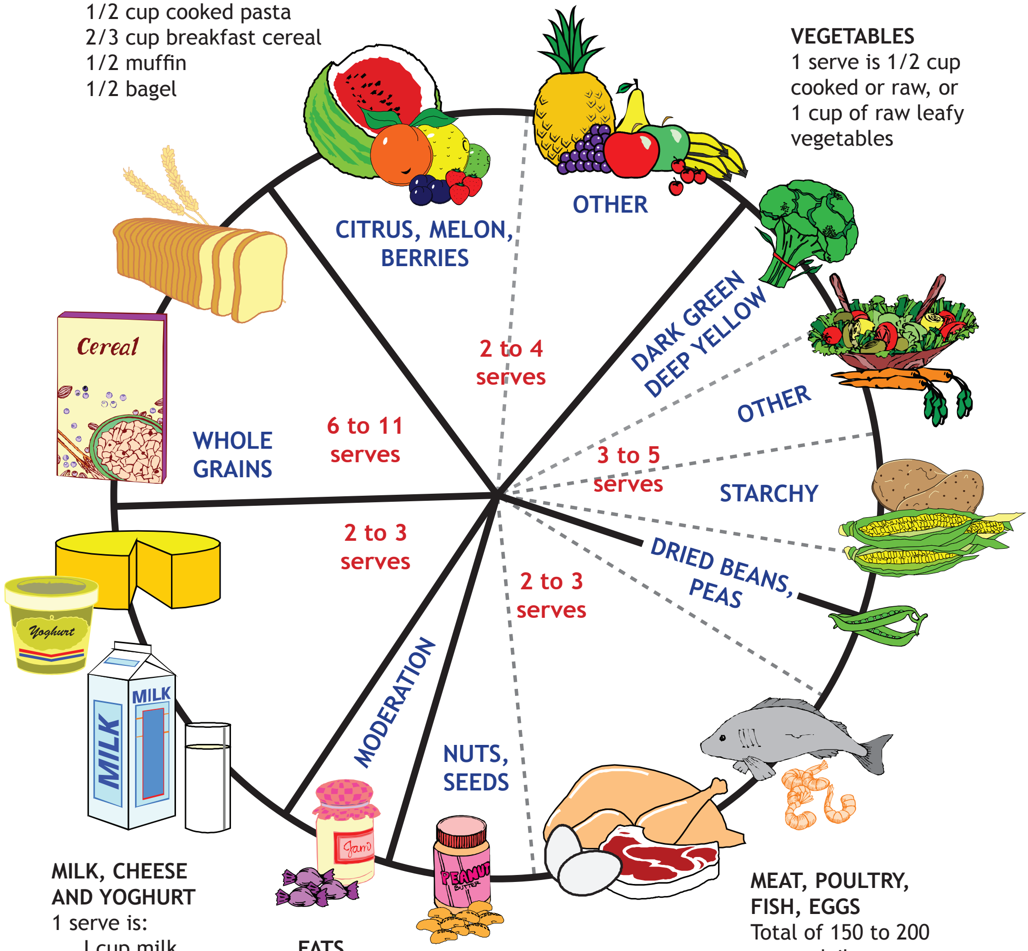

MILK, CHEESE AND YOGHURT
1 serve is:
- I cup milk
- 3cm cube of cheese
- 200 gram carton of yoghurt

FATS, SWEETS
Keep to a minimum

ALCOHOL
None

MEAT, POULTRY, FISH, EGGS
Total of 150 to 200 grams daily

Breastfeeding

Your body is well prepared for feeding your newborn baby. It has stored energy during the middle stages of your pregnancy ready to begin production of breast milk as soon as your baby is born. In fact, you may have already noticed, in the latter part of your pregnancy, a thickish milk leaking from your breasts. Colostrum provides sustenance for your baby for the first three to five days of its life until your breast milk is in full production. The antibodies in breast milk will provide your baby with protection against any infections over the duration of breast feeding.

Breast milk alone is the best food for your baby for the first six months. Your breast milk is specifically suited to your baby's growth needs. At the beginning of a feed your milk is lower in fat, but after you "let-down" the fat content increases. Suckle on demand to ensure the maintenance of an adequate supply to meet growth needs of your baby.

The changes	Special points about nutrition
• Your body needs even more energy to produce milk than to provide nourishment to your unborn child. You may feel tired, hungry and thirsty!	• Eat to your appetite, choosing foods of high nutritional quality. (refer to the 'Food Wheel' on page 10). These foods will provide ALL the vitamins and minerals you need.<br>• Drink well over the day. Eight to ten glasses of juice, milk or water would be adequate.<br>• Rest whenever possible.

Breastfeeding (continued)

The changes	Special points about nutrition
• Your milk is providing the minerals needed for the development of your baby's bones and teeth.	• You require 1200mg calcium to meet this need and maintain your own bone status.<br><br>**For example:**<br>2 ½ cups low fat milk 875mg<br>1 cube cheese 260mg<br>1 orange 90mg<br>½ cup broccoli 80mg<br>TOTAL 1265mg<br><br>See 'calcium pamphlet' available at KEMH.
• Remember that your milk can carry drugs such as alcohol and caffeine across to your baby. It is more difficult for your baby to break these down and expel them.	• Always check with your doctor before taking medications.<br>• Limit your alcohol and caffeine intake. (See pages 1 and 2).
• Weight loss appears to be individual. Some people are less efficient at using energy than others. Watch and adjust your meal intake accordingly.	• For those of you losing weight quickly, eat more foods of high nutritional quality.<br>• If you fail to lose or actually begin to gain weight choose foods high in nutritional quality, and limit your fat and sugar intake. (See pages 3 and 4).
• Maintain the fitness of your body	• Exercise regularly – walking, cycling, swimming are the most appropriate.

Disclaimer: The advice and information contained herein is provided in good faith as a public service. However the accuracy of any statements made is not guaranteed and it is the responsibility of readers to make their own enquiries as to the accuracy, currency and appropriateness of any information or advice provided. Liability for any act or omission occurring in reliance on this document or for any loss, damage or injury occurring as a consequence of such act or omission is expressly disclaimed.

Compiled by: Department of Nutrition and Dietetics

Website: http://wnhs.health.wa.gov.au

© September 2008 WNHS 510

Reviewed July 2010

This information is available in alternative formats upon request

WOMEN AND NEWBORN HEALTH SERVICE

King Edward Memorial Hospital

374 Bagot Road Subiaco WA 6008

Telephone: (08) 9340 2222

Government of Western Australia
Department of Health

Delivering a **Healthy WA**
