# Supplementary material for: Primary prevention of gestational diabetes for women who are overweight and obese: a randomised controlled trial
Source: BMC Pregnancy Childbirth. 2013 Mar 13;13:65. doi: 10.1186/1471-2393-13-65 (PMC3669050; doi:10.1186/1471-2393-13-65)
Supplement: Additional file 3 — Pregnancy Weight Matters pamphlet. Health information pamphlet (provided as usual care to all women booked at Barwon Health) given to women in both trial arms with a reminder/thank-you for returning the questionnaire at 36 weeks. [file 1471-2393-13-65-S3.pdf]

During pregnancy it is normal to gain weight as your baby grows and your body adapts to being pregnant.

However, gaining too much weight or being overweight can cause complications for both you and your baby. These complications can occur while you are pregnant, during labour or after your baby is born.

The purpose of this information is to help you to understand the risk (to you and your baby) of gaining too much weight or being overweight in pregnancy and help you manage your weight gain.

Weight is often a sensitive issue for women. In pregnancy many women struggle with body shape changes and you may feel uncomfortable discussing weight issues with your local health professional.

The following information will help you to understand why additional precautions are taken during pregnancy.

Pregnancy is not a time for strict dieting. However you do not need to eat for two!

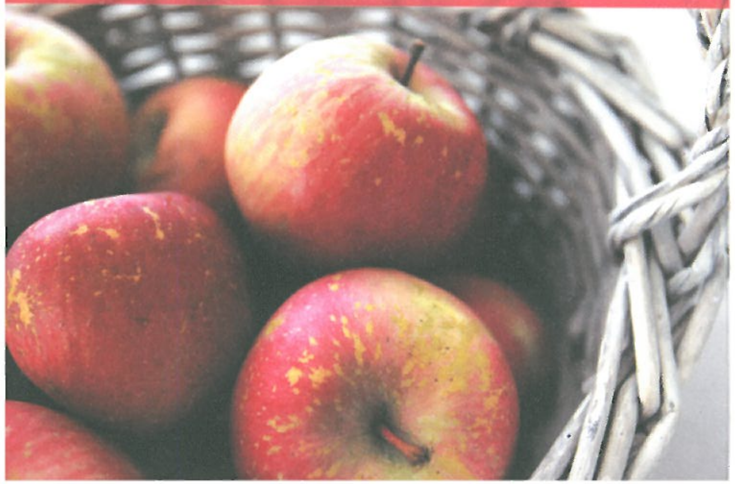

WHO TO CONTACT

For more information about pregnancy weight matters, contact Baxter Maternity Services at Geelong Hospital on T 5226 7511.

References

Further information on pregnancy can be found at:

Royal Women's Hospital
www.thewomens.org.au

Queensland Health Dietitians
www.health.qld.gov.au/masters/copyright.asp

Institute of Medicine Revised Guidelines 2009 can be found at:
www.iom.edu/obect.File/Master/68/230.Report%20Brief%20-%20Weight%20Gain20During%20Pregnancy.pdf

This brochure has been reproduced and adapted by Barwon Health with the kind permission of GV Health.

GV Health /Publication GVH0333

Prepared by the Nutrition & Dietetics Department - For review 2013

OUR VALUES
RESPECT
COMPASSION
COMMITMENT
ACCOUNTABILITY
INNOVATION

Barwon Health

Barwon Health

MATERNITY SERVICES

PREGNANCY WEIGHT MATTERS

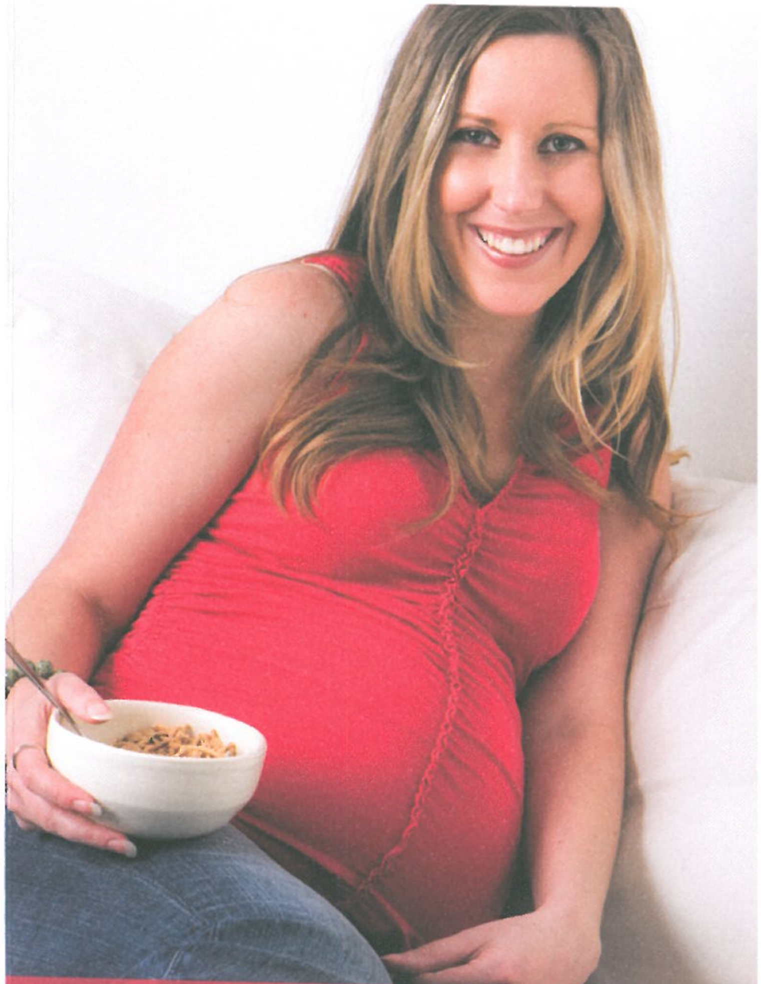

MANAGING YOUR WEIGHT GAIN DURING PREGNANCY

HOW DO WE MEASURE WEIGHT?

The amount of weight you should gain in your pregnancy depends on your pre-pregnancy weight. You need to know your height (without shoes) and weight (in light clothing) to calculate your body mass index (BMI), or your weight adjusted for your height.

On the following graph trace across the line for your height and up for your weight and this will tell you what your BMI is.

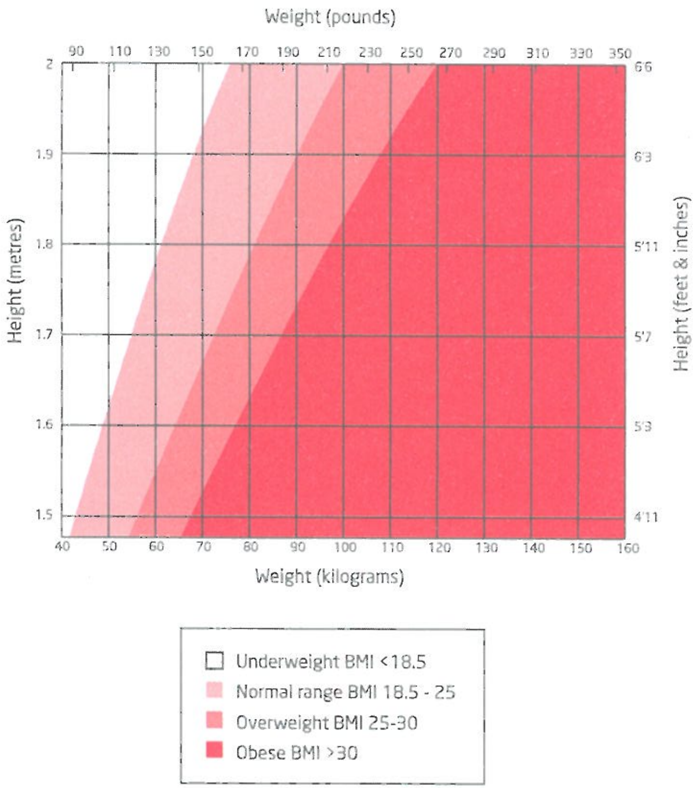

HOW MUCH WEIGHT SHOULD I GAIN IN MY PREGNANCY?

The amount and pattern of weight gain varies for each woman and each pregnancy. The following table is a general guide to expected weight gain. Minimal weight gain is expected in the first trimester of pregnancy.

Expected weight increase per trimester of pregnancy

Trimester	Underweight	Healthy weight range	Overweight	Obese
BMI	Less than 18.5 kg/m²	18.5 - 24.9 kg/m²	25 - 29.9 kg/m²	Higher than 30 kg/m²
First	1 - 3 kg	1 - 3 kg	0 - 1 kg	0 - 1 kg
Second	5 - 7 kg	5 - 6 kg	3 - 5 kg	2 - 4 kg
Third	6 - 8 kg	5 - 6 kg	4 - 5 kg	3 - 4 kg
TOTAL	12 - 18 kg	11 - 16 kg	7 - 11 kg	5 - 9 kg

Institute of Medicine Guidelines 2009

WHAT ARE THE RISKS OF GAINING TOO MUCH WEIGHT DURING YOUR PREGNANCY?

Most pregnancies are uncomplicated. However, gaining too much weight or being over your most healthy weight increases the risk of a number of pregnancy complications. The higher your BMI the more your risk will increase. Increased risk includes:

When you are pregnant

- Gestational diabetes – a form of diabetes that occurs in pregnancy
- Pre-eclampsia – high blood pressure and loss of protein in the urine
- Abnormalities of your baby's growth, development and general health
- Sleep apnoea – a condition that causes you to temporarily stop breathing while you are sleeping

During labour

- Failure of labour to progress
- Shoulder dystocia (the baby's shoulders get stuck during birth)
- Difficulties monitoring the baby's heart
- Difficulties with providing satisfactory pain relief in labour
- Increased risks with attempted vaginal (normal) birth after caesarean section
- Increased need for emergency caesarean section
- Increased risk of complications related to caesarean section

After the birth of your baby

- Increased risk of wound infection
- Increased risk of blood clots (particularly following ceasarean section)
- Increased risk of postnatal depression

WHAT IF I AM GAINING TOO MUCH WEIGHT?

Pregnancy is not a time for strict dieting. However you do not need to eat for two! There are some simple choices you can make that will help you to limit your intake of additional energy and manage your weight gain in pregnancy. Please ask your midwife or doctor for a referral to a dietitian.

Limit the amount of fat you eat

- Reduce your intake of snack foods such as biscuits, cakes, chips, crisps and chocolate
- Reduce the amount of fat or oil used in cooking
- Choose low fat or reduced fat dairy foods such as milk, yoghurt and cheeses. These products still have all the calcium you need for your bones
- Avoid eating cream or sour cream
- Trim all fat off your meat before cooking
- Remove skin from chicken
- Limit high fat take-away foods

Limit high sugar foods

- Drink water not soft drink or cordial
- Limit sweetened soft drinks
- Limit fruit juices to once a day as these are high in natural sugar
- Limit chocolate, lollies and sweets
- Go easy on desserts

Try to minimise snacking, but if you do need to snack, choose options such as fresh fruit, low fat yoghurt, or dry biscuits with reduced fat hard cheese. Try to do as much exercise as you can. Regular exercise can help prevent excess weight gain.
